# Predictive coding is not a theory of anticipation

**DOI:** 10.3389/fnhum.2026.1838057

**Published:** 2026-07-08

**Authors:** Romain Brette

**Affiliations:** Sorbonne Université, CNRS, Institute of Intelligent Systems and Robotics (ISIR), Paris, France

**Keywords:** active inference, anticipation, generative model, neural coding, predictive processing

Anticipation is a hallmark of all living phenomena, from bacteria to humans. If I notice a cloudy sky, I may take an umbrella to avoid getting wet. In cyanobacteria, which are photosynthetic bacteria, the chromosome decompacts shortly before dawn thanks to circadian rhythms, so that gene expression is favored when it is needed (Johnson et al., 2008). To the extent that cognition determines goal-directed behavior, it is fundamentally an anticipatory process (Brette, 2026).

For this reason, predictive coding theory (or predictive processing) has emerged as a popular theoretical framework in neuroscience (Clark, 2013; Rao and Ballard, 1999). It depicts the brain as a “prediction machine,” in which hierarchically arranged “prediction neurons” are tuned to cancel sensory inputs through top-down projections. The remainder, called the “prediction error,” is transmitted to the next layer in the hierarchy. The neural network is then called a “generative model” of the sensory input. The activity of prediction neurons is called the “causes” of the inputs, and the mapping from sensory input to this activity is then “inferring the causes.” In its most ambitious version, predictive processing claims to account for decision making and goal-directed behavior in general thanks to “active inference” (Friston, 2010, 2009), where action is selected so as to minimize prediction error.

Before asking whether the model fits the structure or physiology of the nervous system (see, e.g., Walsh et al., 2020), I suggest starting with a more fundamental question: what is predictive processing a model of? The terminology suggests that predictive coding theory explains how the organism anticipates. However, this terminology is misleading.

First, while predictive coding is regularly presented as being about what happens next (Clark, 2013), the original foundational model of Rao and Ballard (1999) is a model of neural coding of static images: it is not at all about what happens next, since there is no sequence. The “predictions” of neurons are to be understood as “guesses”: one can guess what image was presented by observing the activity of those neurons. But the activity of neurons always follows, not precedes, the presentation of images. This confusion is especially manifest in the fact that the activity of “prediction neurons” is alternatively or simultaneously depicted as representing both the “causes” of the inputs and the predictions, for example:

“*A non-trivial assumption of predictive processing is that the brain seeks to predict the causes of its observations*.” (Kirchhoff, 2018)

It is certainly non-trivial to predict a cause. The reason for this peculiar turn of phrase is that, in the original predictive coding literature, the term “prediction” simply means that an output is being compared to something else, but does not refer to an anticipation of future events. The same holds for spike-based predictive coding models (Boerlin et al., 2013), which implement greedy algorithms. There have been a few subsequent attempts to extend these models to the temporal domain (Rao et al., 2023; Wacongne et al., 2012), but those are rather different models—for example, the model of Rao and Ballard (1999) calculates its optimal “prediction” by a gradient descent while the input is held constant. Thus, predictive coding theory is, as initially construed, an efficient coding theory, not a theory of anticipation.

Yet, this original work continues to be presented as the main neural model of predictive processing and active inference. For example, a recent review of active inference writes:

“*Regarding neural implementation, one of the most widely entertained hypotheses*—*about how the brain might implement perceptual inference*—*is predictive coding (Rao and Ballard, 1999)*.” (Pezzulo et al., 2024)

If this is true, then perceptual inference is not in fact conceived as anticipatory. In the same way, a recent book on active inference and the free energy principle writes:

“*Under some assumptions, the dynamics that emerge from generative models used in Active Inference closely correspond to widespread models in computational neuroscience, such as predictive coding* (*Rao and Ballard, 1999).”* (Parr et al., 2022)

Second, even assuming that the predictions of predictive coding theory are amended to be about the future and not the past, we may wonder how canceling the next sensory input relates to anticipatory behavior. The prediction of a predictive coding network is indeed what gets subtracted from the sensory input, and it succeeds to the extent that it matches the input. This prediction is not about a meaningful category of events: it is a numerical prediction (a vector) of the sensory input pattern at a particular time. It is doubtful that this kind of prediction underlies a significant part of anticipatory behavior. Consider the umbrella example: I notice clouds, I predict that I might get wet, unless I take an umbrella. The prediction is not numerical, it is not timed, it is conditional, and if I act accordingly, it will not happen. Consider posture: the runner anticipates the start of the race by placing herself in starting blocks. There is nothing to be canceled here, just an action that will benefit the runner in the future. Consider object permanence: if someone disappears behind a tree, we expect that turning around the tree will make her visible again. We do not expect a particular visual pattern, and the prediction is conditional on a specific but untimed action. Consider affordances (Gibson, 1979): we see a chair and we anticipate that we can sit on it. Again, this is not a numerical prediction of the next pattern of tactile stimulation. Predicting the consequences of hypothetical actions can be useful; predicting the next sensory pattern is less obviously so.

But a predictive coding network allegedly implements a model of the world, namely a generative model, and surely a model of the world is a useful thing to have. However, a generative model is a very peculiar kind of model, unlike all models used in physics. A generative model is a mapping from a lower-dimensional vector (the activity of higher-order neurons) to the target domain (e.g., images). In contrast, models of physics typically express relationships between measurable quantities, such that one can anticipate the effect of a manipulation: for example, if I compress a gas, its pressure will increase. It is not so useful, on the other hand, to generate an instance of the positions and velocities of all particles in a gas from a lower-dimensional description.

Again, terminology is misleading. When it is claimed that the predictive coding network infers the “hidden causes” of the sensory input thanks to the generative model it implements, what this means concretely is that the activity of higher-order neurons maps to the sensory input. In the same sense, a Fourier transform also “infers the causes” of the image. Here, “cause” is simply statistical jargon to mean a parameter value, and “inferring the causes” is the process of fitting the parametric model, with no causation actually involved. Specifically, the fitted model is a Bayesian model of sensory inputs, and the values of its variables are inferred. This amounts to answering the question “How does the world work?” by “42,” and this is hardly a model of the world (Brette, 2019).

At this point, predictive coding theory is a theory of neural coding (specifically, efficient coding) implementing statistical inference, not a theory of anticipation. *Active inference* (Friston, 2009; Nave, 2025) then claims to address anticipatory behavior by seeing action as a special case of statistical inference:

“*This allows one to frame goals and preferences in terms of prior beliefs, such that goals are subsequently fulfilled by action […]. This furnishes an explanation of behavior in terms of one straightforward imperative—to minimize surprise or, equivalently, to maximize Bayesian model evidence*.” (Friston et al., 2015)

In other words, you take your umbrella because you expect to remain dry. Thus, predictions are not simply used to select the appropriate action to achieve the organism's goals; they *are* the goals. Actions are selected so as to meet the organism's prediction. This provocative idea has some superficial appeal, since it immediately dissolves the difficult problems of action selection and goal-driven behavior.

But why then do we not simply go to the closest dark room and remain there, forever unsurprised? The reply to the dark room objection is straightforward: we do not go to the next dark room because we predict that there will be light (Friston et al., 2012), a rhetorical trick that Bickhard aptly calls “epicycles” (Bickhard, 2016)—anything that does not fit the theory can simply be an extra assumption called “prior.” In reality, the dark room objection still stands if learning is allowed, since the organism will progressively move to the more predictable environment, whatever the initial prior (Brette, 2026).

But in any case, there is a fundamental reason why choosing the action that meets the sensory prediction cannot be a valid principle: at any given time, many predictions can be made. Perhaps the runner predicts that she will win the race, and thereby places herself in the starting blocks so as to “minimize surprise.” But she might also notice clouds and predict that, with an umbrella, she will not get wet. Should she take an umbrella instead? More modest runners might predict that they will lose the race. Will they just stand by and watch the conceited runner win, since they are supposed to make their prediction happen? This should go without saying, but to predict and to want are actually two different things: you do not expect to be dry, you *want* to be dry, and you predict that you will be dry if you take your umbrella, and that you will be wet if you do not take your umbrella (two opposite predictions, one goal).

Proposing that action is selected so as to meet the organism's prediction means that the current goal gets called “prediction,” while all other predictions are ignored. If this is just a renaming game, then what active inference proposes is that the action is selected so as to fulfill the organism's goal. In other words, the theory has exactly no explanatory content. It also erases the actual relevance of predictions for action selection: predicting the consequences of a set of options allows picking the right option; predicting the future like an Oracle does not.

This framework does not account for anticipatory behavior. Despite its suggestive name, predictive coding theory is not a theory of anticipation. It is a neural coding theory. The anticipatory decompaction of the cyanobacterium's chromosome before dawn is mediated by an oscillatory physiological network entrained by sunlight, not by the cancellation of light input. Is anticipation even necessarily mediated by predictions? Cyanobacteria do not speak, but we might want to say that the chromosome's compaction *predicts* dawn. However, this is a metonymic use of “prediction,” as in “low atmospheric pressure predicts rain.” It is the climate scientist who actually predicts rain based on pressure; the climate does not actually predict anything. The relevant part of the biological phenomenon, which deserves the term “anticipation,” is not that some physiological quantity correlates with some future quantity—this is already the case of the light input. It is the fact that the cell's physiology appears to exploit the regularity of the day-night cycle to maximize photosynthesis. Anticipation means exploiting regularities, not necessarily uttering prophecies.
